# Pulmonary Actinomycosis Mimicking Pulmonary Cancer on Fluorine-18 Fluorodeoxyglucose PET-CT

**DOI:** 10.7759/cureus.12306

**Published:** 2020-12-26

**Authors:** Shota Tanaka, Hisatoshi Araki, Takeshi Yoshizako, Hajime Kitagaki, Takeshi Isobe

**Affiliations:** 1 Department of Radiology, Shimane University Faculty of Medicine, Izumo, JPN; 2 Department of Internal Medicine, Division of Medical Oncology & Respiratory Medicine, Shimane University Faculty of Medicine, Izumo, JPN

**Keywords:** pulmonary actinomycosis, pulmonary cancer, pet-ct, ct imaging

## Abstract

Pulmonary actinomycosis is a rare disease frequently misdiagnosed as primary lung cancer. Herein, we presented pulmonary actinomycosis mimicking pulmonary cancer on fluorine-18 fluorodeoxyglucose (F-18-FDG) PET-CT.

## Introduction

Pulmonary actinomycosis is a rare disease that is frequently misdiagnosed as primary lung cancer or as other more conventional lung infections. It often shows positive uptake on fluorine-18 fluorodeoxyglucose (F-18-FDG) PET-CT and is only diagnosed by cytological/histological examination. We report a case of pulmonary actinomycosis mimicking lung cancer on F-18-FDG PET-CT with a review of the literature.

## Case presentation

A 60-year-old man had left-sided chest pain during inspiration after being hospitalized with cerebral infarction. There was a slight fever of 37°C from the time of admission, and there was a slight increase in peripheral leukocytes and c-reactive protein. No respiratory symptoms other than chest pain during inspiration. There was no pleural friction rub or crackles. No weight loss was observed. Past history includes type 2 diabetes, hypertension, hyperuricemia, hypertrophic cardiomyopathy, and paroxysmal atrial fibrillation. The patient smoked 50 pack-year and was a moderate drinker. There was no dental disease.

**Figure 1:**
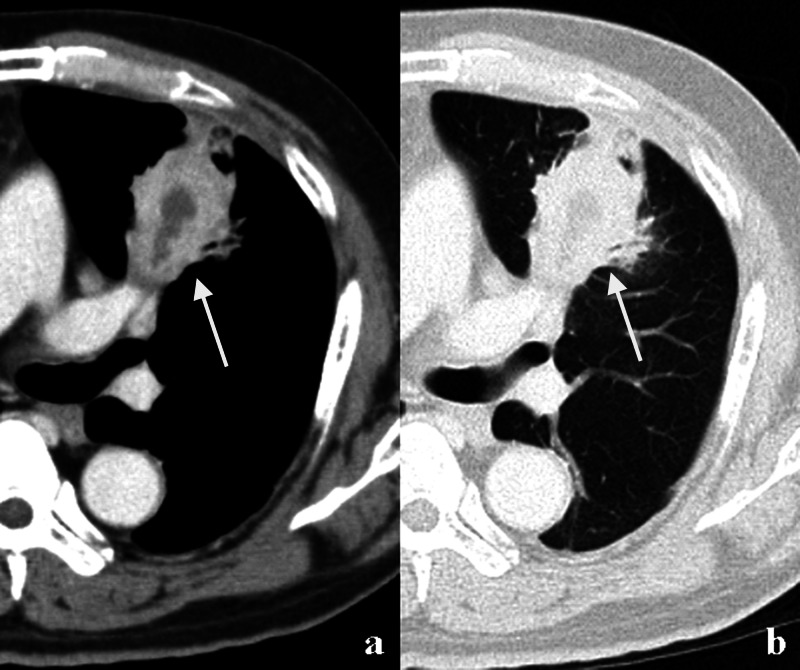
Enhanced chest CT The enhanced chest CT showed a 4.0 cm mass-like lesion in the lingular segment of the left lung. (a) Soft tissue density image and (b) lung density image. There was slight pleural thickening, and it was suspected that inflammation had spread to the pleura.

The chest X-ray film taken on admission revealed a large mass in the left middle lung field. Chest CT showed a 4.0 cm mass-like lesion in the lingular segment of the left lung. The mass-like lesion caused segmental consolidation and contained low-attenuation areas with peripheral enhancement on enhanced CT (Figure 1).

**Figure 2:**
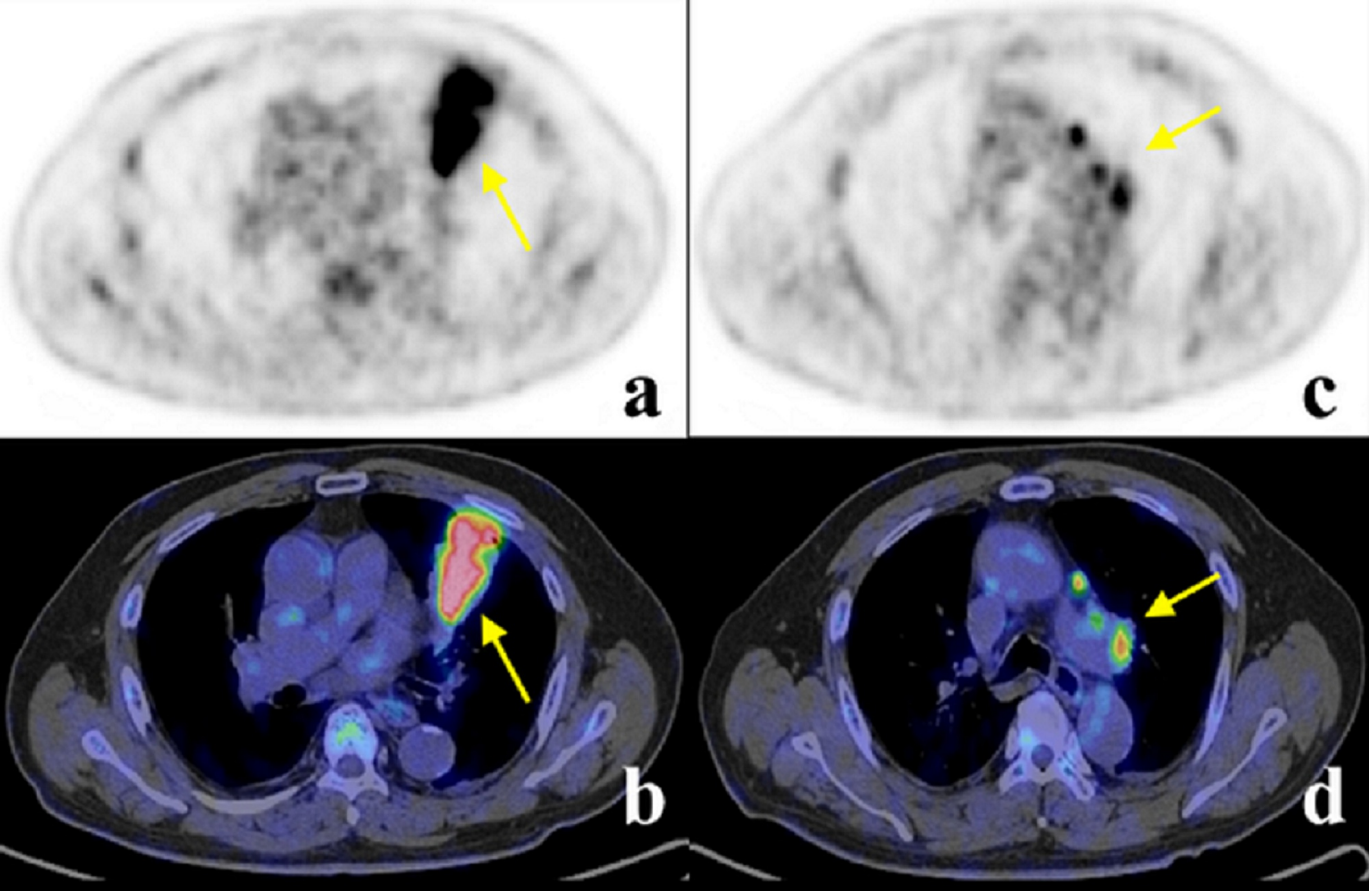
The F-18-FDG PET-CT The F-18-FDG PET-CT showed a high uptake around the mass and the lymph nodes. (a) Tumor level PET; (b) tumor level PET-CT; (c) lymph nodes level PET; (d) lymph nodes level PET-CT. F-18-FDG: fluorine-18 fluorodeoxyglucose

Biochemical studies showed a slight elevation of carcinoembryonic antigen (CEA: 6.1 ng/ml, normal range 4.0). It was initially suspected to be complicated with lung cancer and obstructive pneumonia. F-18-FDG PET revealed high metabolism (early maximum standardized uptake value {SUV} of 9.5 and delayed maximum SUV of 12.1) by the mass-like lesion and also in lymph nodes (maximum SUV of 5.7) (Figure 2).

We first suspected lung cancer, performed a CT-guided biopsy, and bronchoscopy. The biopsy specimen showed basophilic and lobulated bacterial mass by HE staining, and inflammatory granulation tissue was collected around it. Grocott's staining was positive. Ziehl-Neelsen staining was performed but was negative. No findings suspected of being malignant are found. *Actinomyces israelii* identified in bronchial lavage fluid and sputum cultures.

**Figure 3:**
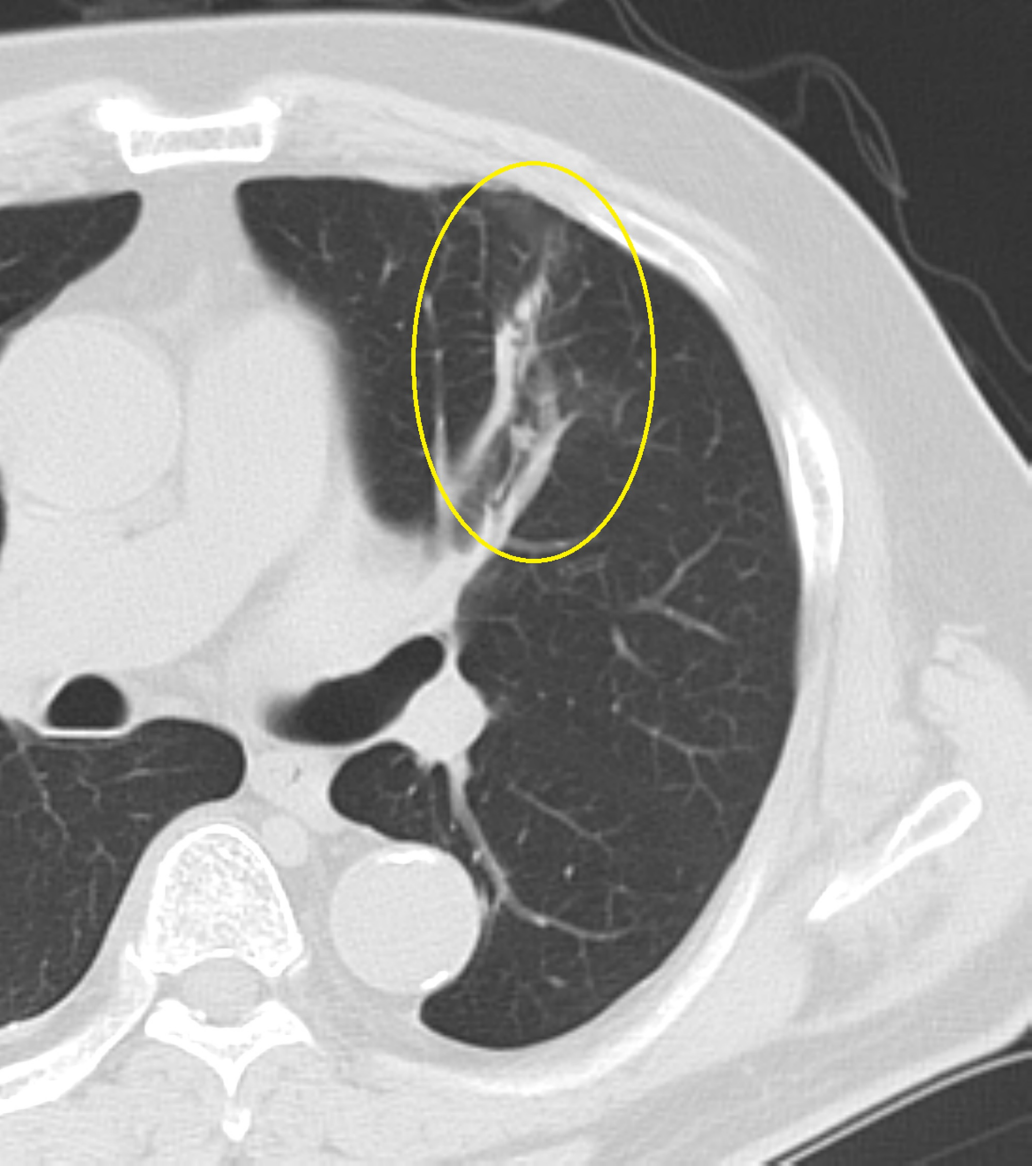
Chest plain CT taken one year later One year later, CT showed that the mass-like lesion became a cord-like shadow.

Initially, we administered tazobactam/piperacillin intravenously for the first two weeks, and after it was found to be actinomycete, we treated it with oral penicillin. The administration was continued until five months after the improvement of shadow was observed by follow-up Imaging. Even after the administration was completed, the shadow keeps shrinking and became a cord-like shadow, and the patient improved without complications (Figure 3). No surgical treatment is performed.

## Discussion

Actinomyces are facultatively anaerobic Gram-positive, nonspore-forming, prokaryotic bacteria with a peculiar fungus-like morphology. Actinomyces normally colonize the oropharynx, gastrointestinal tract, and genital mucosa, but can affect any organ of the body. Pulmonary actinomycosis is a rare condition that mainly involves the lung parenchyma. It is commonly acquired through the aspiration of microorganisms from oropharyngeal or gastrointestinal secretions, as well as by inhalation, by hematogenous dissemination, and by direct extension from adjacent infected tissues. Poor oral hygiene, dental problems, gastroesophageal reflux disease, oral trauma, infections, chronic debilitating diseases, and other conditions predispose patients to pulmonary actinomycosis [1].

The typical CT features of parenchymal actinomycosis are a region of chronic segmental consolidation containing necrotic low-attenuation areas with frequent cavity formation. A broncholith can be secondarily infected by Actinomyces, resulting in endobronchial actinomycosis. This usually manifests as proximal endobronchial calcification associated with distal obstructive pneumonia [2]. Though a broncholith was not found in this patient, the pulmonary parenchymal findings on CT were characteristic of the disease.

The main feature of actinomycosis on F-18-FDG PET-CT is intense uptake (maximum SUV of 5.4-13.7), resembling malignancy [3-5]. In our patient, the actinomycosis-related elevation of the SUV was probably due to acute inflammation involving all layers of the bronchial mucosa and extension into the lung parenchyma. Pulmonary actinomycosis is often diagnosed as a result of the pathological examination after surgery. Our case showed that CT findings are equivalent to F-18-FDG PET findings regarding the characteristics of this disease.

## Conclusions

It is important to consider specific inflammatory diseases like pulmonary actinomycosis when a mass lesion shows a very strong uptake on F-18-FDG PET.
